# Value-based attention capture: Differential effects of loss and gain contingencies

**DOI:** 10.1167/jov.20.5.4

**Published:** 2020-05-12

**Authors:** Mark W. Becker, Samuel H. Hemsteger, Eric Chantland, Taosheng Liu

**Affiliations:** 1 Department of Psychology, Michigan State University, East Lansing, MI, USA; 2 Department of Psychology, Michigan State University, East Lansing, MI, USA; 3 Department of Psychology, Michigan State University, East Lansing, MI, USA; 4 Department of Psychology, Michigan State University, East Lansing, MI, USA

**Keywords:** value-based attention, visual attention, loss, attention capture

## Abstract

There is evidence that attention can be captured by a feature that is associated with reward. However, it is unclear how associating a feature with loss impacts attentional capture. Some have found evidence for attentional capture by loss-associated stimuli, suggesting that attention is biased toward stimuli predictive of consequence, regardless of the valence of that consequence. However, in those studies, efficient attention to the loss-associated stimulus reduced the magnitude of the loss during training, so attention to the loss-associated stimulus was rewarded in relative terms. In Experiment 1 we associated a color with loss, gain, or no consequence during training and then investigated whether attention is captured by each color. Importantly, our training did not reward, even in a relative sense, attention to the loss-associated color. Although we found robust attentional capture by gain-associated colors, we found no evidence for capture by loss-associated colors. A second experiment showed that the observed effects cannot be explained by selection history and, hence, are specific to value learning. These results suggest that the learning mechanisms of value-based attentional capture are driven by reward, but not by loss or the predictability of consequences in general.

## Introduction

Attention is required for the conscious representation of object identities (Lamme, 2003; Mack & Rock, 1998; Rensink, O'Regan, & Clark, 1997), yet the number of items that can be simultaneously attended is exceedingly small (Becker & Pashler, 2005; Pashler, 1988; Pylyshyn & Storm, 1988). As a result, it would seem important to have mechanisms that ensure that attentional resources are devoted to important aspects of the environment, rather than being squandered on the irrelevant. Indeed, research suggests that there are a number of mechanisms that help guide the allocation of attention to relevant aspects of the scene. Some of these mechanisms are relatively automatic or “bottom-up,” while others are volitional or “top-down,” and these two systems appear to interact in the competition for attention (Corbetta & Shulman, 2002). Among factors that guide attention, there is evidence that people can set attentional control settings that bias attention to objects that share features of a target object (Folk, Remington, & Johnston, 1992), and people can use knowledge of likely locations for a target to prioritize those locations for attention (Chun & Jiang, 1998; Oliva & Torralba, 2001; Torralba, Oliva, Castelhano, & Henderson, 2006). These findings suggest that multiple factors help ensure that attention is allocated efficiently.

Recently researchers have found that reward-based learning can also prioritize features for attention (Anderson, Laurent, & Yantis, 2011a; Anderson & Yantis, 2012). This work demonstrates that associating a particular feature with high reward leads to “value driven attentional capture” – the rewarded feature automatically captures attention even when it is irrelevant to volitional goals (Anderson et al., 2011a; Le Pelley, Pearson, Griffiths, & Beesley, 2015). The common method of producing value-based attentional capture in the lab (e.g., Anderson et al., 2011a) is to have participants perform a training phase in which one color is associated with high reward and a second color is associated with low reward. During a subsequent test phase, color is made irrelevant to the task, and participants must search an array of objects for one unique shape—a shape singleton task. During this task, one of the distractor items occasionally appears in one of the previously rewarded colors. The typical finding is that the high-reward distractor is effective at capturing attention, thereby delaying the time to find the shape target (Anderson & Yantis, 2012, 2013; Bucker, Belopolsky, & Theeuwes, 2015; Mine & Saiki, 2015; Müller, Rothermund, & Wentura, 2015). The low reward distractor may also capture attention, but to a lesser extent (Anderson, 2013).

This value driven attentional capture phenomenon has garnered a great deal of recent interest for two reasons. First, from a basic science perspective, it provides insight into a basic learning mechanism that helps ensure that attention is biased toward relevant aspects of the environment. Second, at a practical level, value driven capture may have implication for understanding one of the factors that maintains addiction. Addiction is associated with an attentional bias toward stimuli associated with the addiction (Lubman, Peters, Mogg, Bradley, & Deakin, 2000; Marissen et al., 2006; Robbins & Ehrman, 2004), and this attentional bias is believed to be one factor that contributes to relapse among those seeking to quit their addiction (Field & Cox, 2008). According to this theory, the attentional bias toward addiction related stimuli results in conscious processing of those stimuli, thereby increasing craving and leading to relapse (Robinson & Berridge, 2008).

To date, the work on value-driven capture has shown that associating a feature with reward leads to a robust capture of attention by that feature, even when it is irrelevant and harmful to one's task performance (Anderson et al., 2011a; Le Pelley et al., 2015). However, the attentional effect of associating a feature with loss is less clear.

Two studies that have used methods following the original value-based capture paradigm have concluded that stimuli associated with both gain and loss capture attention and do so to a similar extent (Wang, Yu, & Zhou, 2013; Wentura, Müller, & Rothermund, 2014). However, the methods in these studies did not actually associate attending to the punished color with loss. Instead, the color was associated with the possibility of experiencing a substantial loss, but attending to the color and rapidly responding could eliminate or reduce this loss. For instance, in Wentura, Muller, and Rothermund (2014), participants would lose 20 points if they responded incorrectly or too slowly, but would lose nothing if they responded rapidly and correctly. Similarly, in Wang, Yu, and Zhou's Experiment 1 (2013), participants would lose 15 points if they responded too slowly or incorrectly to the punished color and would lose only 10 points for responding quickly and accurately. Thus in both experiments the optimal strategy was to learn to rapidly attend to the punished color. That is, in relative terms attending to the punished color was actually rewarding. In behaviorist terms, these training protocols reinforced attention to the “punished” color; the only difference between the rewarded and punished colors was that attention to the rewarded colors was positively reinforced, while attention to the punished color was reinforced via negative reinforcement (the removal/reduction of a negative outcome). To our knowledge there are no studies that have paired a feature with a financial loss that avoided such potential confounds (but see Schmidt, Belopolsky, & Theeuwes, 2015 for a study of classical fear conditioning to a feature). Thus, although these studies are informative in showing that value-based capture can be driven by negative reinforcement, they do not address how punishing attention to a feature (by presenting a loss or negative outcome) impacts the attentional priority of that feature.

To further investigate this issue, here we use a paradigm similar to that used in previous studies showing that loss-associated stimuli capture attention, but we no longer reinforce attending to the loss-associated stimuli. Instead, we make it optimal to suppress attention to loss-associated stimuli, to determine whether doing so would still lead to capture of attention by that feature.

## Experiment 1

The method in Experiment 1 followed the value capture paradigm of Anderson and Yantis (2011a), but rather than having one color associated with high reward and the second with low reward during training, we had one color associated with reward, a second associated with loss, and a third that had no reward contingency. During the test phase, there were four conditions; three in which the target-colors from the training phase appeared as distractors, and a fourth in which a color that had not been associated with a target during training appeared as a target. The condition where the distractor was a prior target color that was not associated with either reward or loss is an appropriate baseline for evaluating the capture of attention, as it controls for possible attentional capture by the color simply being a target during training (Sha & Jiang, 2015). The second control condition, which had no training-relevant distractor in the test phase, is similar to what prior studies used and is presented for comparison with those studies.

## Methods

### Participants

Forty-eight undergraduates who reported normal or corrected-to-normal vision, including color vision, participated for financial compensation. This sample size was based on a G*Power (Faul, Erdfelder, Lang, & Buchner, 2007) calculation of the sample size that would yield >0.9 power to detect an effect size of 0.2 (Cohen's effect size f) for the omnibus analysis of the test phase (a univariate repeated-measures analysis of variance [ANOVA] with four levels). Prior research suggests that the effect size for attentional capture is usually >0.3 (e.g., Anderson & Yantis, 2012; Gupta, Hur, & Lavie, 2016; Müller et al., 2015; Wang et al., 2013). We wanted to be conservative in our approach, thus we chose a smaller effect size when determining our sample size. Participants gave informed consent and the experimental procedures were approved by the Michigan State University Institutional Review Board and adhered to the tenets of the Declaration of Helsinki.

#### Stimuli

**Figure 1.**
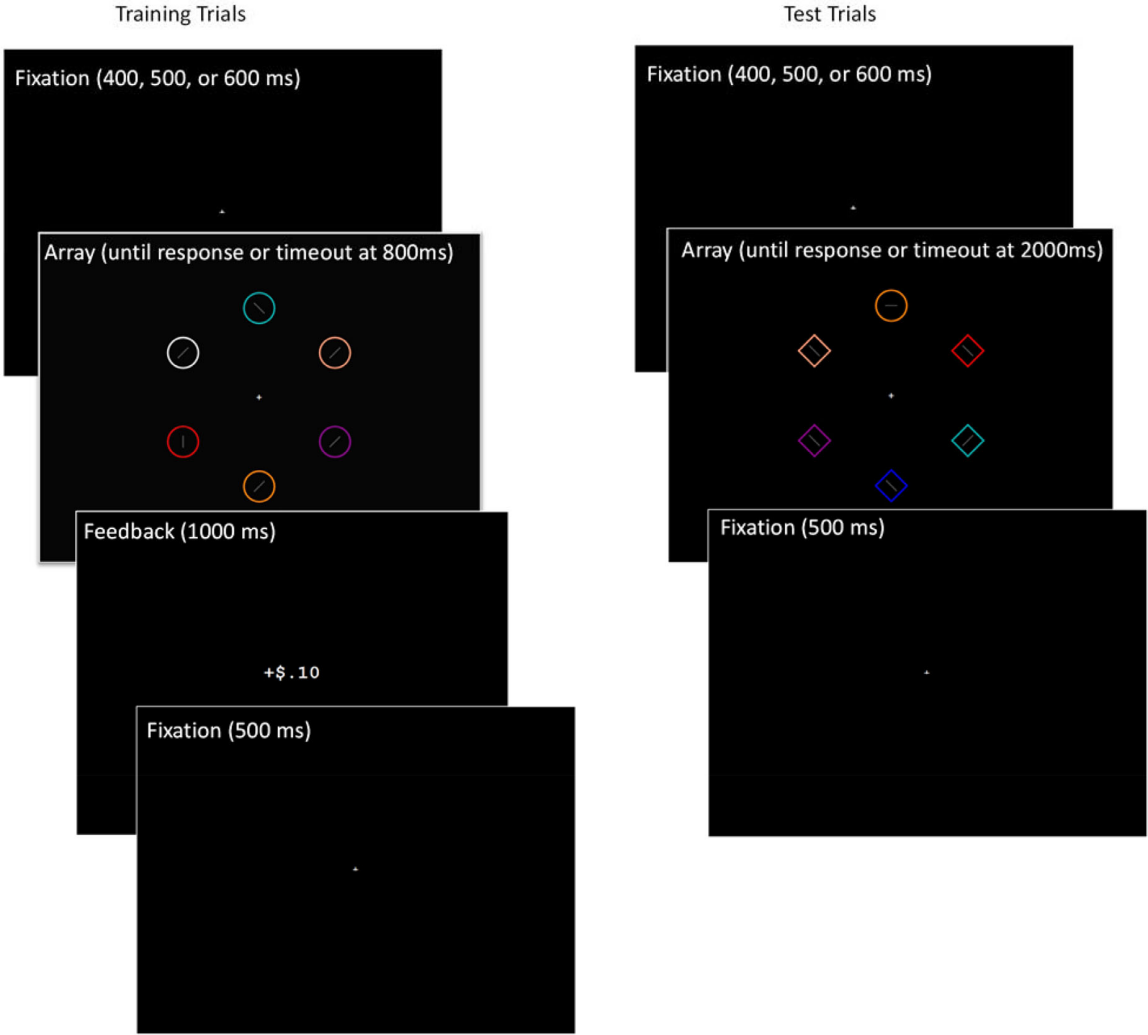
Trial schematic for Experiment 1. The training example represents a trial where a correct response to the red target was rewarded. The test example has the rewarded red item as a distractor.

The experiment was programmed in E-Prime 2.0 (Psychology Software Tools, Sharpsburg, PA, USA). Visual stimuli consisted of diamond- and circle-shaped outlines (size 2.7° × 2.7°, line thickness 0.15°) and line segments (length 1°, line thickness 0.08°) presented on a black background. The shape outlines (referred to as “shapes” for brevity below) could appear in nine possible colors: red, green, yellow, blue, cyan, orange, pink, purple, or white (see Figure 1 for example colors). These colors were selected by the experimenters to be maximally distinct, and followed previous work on value-based attentional capture (Anderson et al., 2011b; Anderson & Yantis, 2012). The line segments were always gray and could be in one of four possible orientations: vertical, horizontal, and the two 45° tilted orientations between the vertical and horizontal. On each trial, six shapes were presented at evenly spaced locations (60° apart) at an eccentricity of 8.9° (Figure 1). Stimuli were presented on 19" cathode-ray tube (CRT) monitors with a refresh rate of 85 Hz and a resolution of 1024 × 768. Participants viewed the screen from approximately 55 cm away in dark, sound-attenuated booths.

### Training and test phases

There were two phases in the experiment: training and test. During training, six circles appeared in different colors with a line segment in each circle. Targets were defined as three colors: red, green, or yellow. On each trial, one target color appeared and the remaining five colors were randomly sampled without replacement from the remaining six non-target colors. Inside the target circle was a vertical or horizontal line segment, whereas inside the nontarget circles the line segments were randomly tilted 45° to both directions. Participants were asked to find a target color and report via button press whether the line segment within that target was oriented horizontally or vertically. Their responses were either rewarded or punished (see below for details).

During the test phase, the stimulus array contained either five circles and one diamond, or five diamonds and one circle. All shapes had different colors and the target was defined as the unique shape. The target shape was never in one of the training phase's target colors, whereas one of the distractors could be in one of the target colors. Again, a line segment appeared in each shape, with tilted lines in the non-target shapes and a vertical or horizontal line in the target shape. Participants were told that color was no longer relevant, and there would be no reward or punishment, and their task was to find the uniquely shaped item in the array and report the line orientation within that shape.

### Design and procedure

Before the training phase started, participants completed 12 practice trials of the training task (see Figure 1 for trial schematic), where the search array was presented for 2000 ms. Participants were given feedback at this time; their response was followed by “Correct,” “Incorrect,” or “Too slow” (if they did not respond within the 2000 ms window).

After the practice, each participant started with $10 in their bank, and they were told that they could earn or lose money on each trial. For these training trials, one of the target colors (red, green, yellow) was assigned to be rewarded, the second was punished, and the third was associated with no reward contingency. The mapping of the reward contingency to target colors was counterbalanced across participants. Participants reported the orientation of the line segment inside the target colored circle. A correct response to a rewarded target was followed by a feedback display that presented “+0.10” (80% of the time) or “−0.10” (20% of the time) in the center of the screen. This payout contingency was reversed for correct responses to targets of the punished color, that is, 80% of trials were followed with a “−0.10” and 20% of trials were followed with “+0.10”. When the target appeared in the no-contingency control color, there was no reward or loss (i.e., no visual feedback was displayed). For all conditions, an incorrect response resulted in a beep sound with no reward, or loss. The search array was displayed until response or for 800 ms, whichever occurred earlier. This short response window is in line with prior experiments which used either 600 ms (Anderson, Laurent, & Yantis, 2011a, Anderson, Laurent, & Yantis, 2011b) or 1000 ms (Anderson & Yantis, 2012). If participants did not respond within 800 ms (timed out), the words “Too Slow” appeared on the screen, and there was no reward or loss. In this way, attention to the punished color was truly punished; rapidly attending and responding to the punished color was associated with loss. Participants completed five blocks of training trials, with each block consisting of 120 trials (40 for each colored target), for a total of 600 training trials. In addition to the trial-by-trial feedback about the reward or punishment, their bank total was displayed during each break between blocks.

During the test phase, participants were told that color was no longer relevant to the task, and their task was to find the unique shape, and report the orientation of the line segment within the unique shape. There were four randomly interleaved conditions. In the Control condition, none of the target colors from the training phase appeared in the display. In the No-Contingency condition, one of the distractors appeared in the target color that was associated with neither reward nor loss during the training phase. In the Rewarded condition, one of the distractors appeared in the rewarded color, and in the Punished condition, one of the distractors appeared in the punished color. The test phase started with 10 practice trials (only the control condition) followed by a single block of 240 trials (60 trials per condition). During the test trials all reward contingencies were removed. Participants were instructed to respond as fast as possible while maintaining accuracy. Following prior work using this task (Anderson, Laurent, & Yantis, 2011a, Anderson, Laurent, & Yantis, 2011b; Anderson & Yantis, 2012), participants were given a longer time to respond during the test block than the training block. In our case, test trials timed out after 1200 ms. At the completion of the experiment, participants were given an open-ended question to query whether they became aware of the reward contingencies during the experiment. The experiment took about 75 minutes to complete.

## Results

We eliminated participants who performed poorly during training in the experiment. Poor performance is defined as less than 50% accuracy for the rewarded stimulus in the last four blocks of the training phase. This criterion led to the elimination of two participants, both from the attend green condition. Thus our final sample had 14 participants in the attend green condition and 16 in each of the attend red and attend yellow conditions. For these remaining 46 participants, we calculated the accuracy and median RTs for each condition. We performed omnibus repeated measure ANOVAs, followed by planned paired *t*-tests to isolate the source of the effects. When paired comparisons failed to reject the null hypothesis, we additionally conducted Bayesian t-tests to evaluate the strength of evidence for the null hypothesis using SPSS. The null hypothesis was that the two conditions had the same mean, and the priors were set to be uniform distributions. We report the Bayes factors in terms of the likelihood of the null over the alternative hypothesis (BF_01_). This method is more intuitive when trying to evaluate support for the null hypothesis as BF_01_ indicates how much more likely the null hypothesis is than the alternative hypothesis. By convention, a Bayes factor of one to three is considered weak evidence for the null hypothesis, three to 20 is considered positive evidence for the null hypothesis, and 20 to 150 is considered strong evidence for the null (Rafterty, 1995; Wagenmakers, 2007).

### Training phase

**Figure 2.**
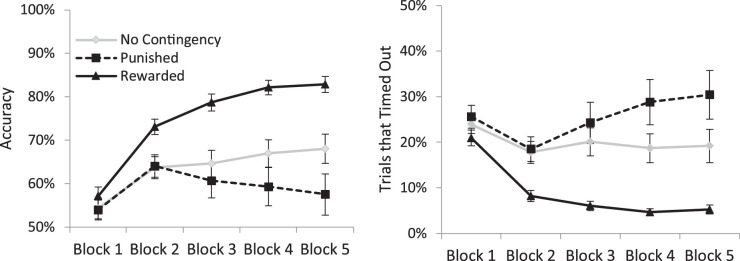
The left panel shows the rate of successful target identifications in the training phase of Experiment 1 as a function of target type and training block. The right panel shows the percentage of trials in which responses were not made within the 800-ms response window as a function of target type and block. Error bars represent the standard error of the mean.

Accuracy for each participant was calculated for each target condition for each of the five training blocks (see Figure 2). A repeated-measures ANOVA with three levels of target type (no contingency target, rewarded target, punished target) and five levels of block found a main effect of target type (F(2,90) = 18.02, *p* < 0.001, η_p_^2^ = 0.286), a main effect of block (F(4, 180) = 24.30, *p* < 0.001, η_p_^2^ = 0.351), and a target type by block interaction (F(8, 360) = 8.74, *p* < 0.001, η_p_^2^ = 0.163). The source of the interaction is clear from the figure and was verified by paired *t*-tests. The three target types did not differ during block 1 (all t(45) < 1.51, *p* > 0.138, BF_01_ > 2.92). Performance in the rewarded target condition was significantly better than the other two conditions in the second block (both t(45) >3.69, *p* < 0.002, d > .599), and these significant differences maintained for all subsequent blocks (all t(45) > 4.2, *p* < 0.001, d > 0.8). The punished target did not differ from the neutral target in the first three blocks (all t(45) <1.23, *p* > 0.22, BF_01_ > 4.2) but marginally worse in the fourth (t(45) = 1.97, *p* = 0.055, d = 0.294) and was significantly worse in the fifth block (t(45) = 2.39, *p* = 0.021, d = 0.376).

The high performance in later blocks for the reward targets, relative to the other targets was due largely to decrease in trials that timed out in the rewarded condition (see Figure 2). An ANOVA on these timed-out trials mirrored the results of overall accuracy. There were main effects of block (F(4,180) = 6.12, *p* < 0.001, η_p_^2^ = 0.12), target type (F(2, 90) = 16.10, *p* < 0.001, η_p_^2^ = 0.26), and a block by target type interaction (F(8, 360) = 7.78, *p* < 0.001, η_p_^2^ = 0.15). Paired *t*-test showed that the rewarded target produced fewer timed-out trials than both the no contingency and punished targets by the second block of trials that maintained throughout the remaining blocks (all t(45) > 3.84, *p* < 0.001, d > 0.72). The punished targets were not significantly different from the neutral until the fourth block (t(45) = 2.38 *p* = 0.022, d = 0.357), and maintained for the fifth block (t(45) = 2.41, *p* = 0.02, d = .36). Given the high number of timed-out trials, we did not attempt to analyze reaction times for the training phase.

### Test phase

**Figure 3.**
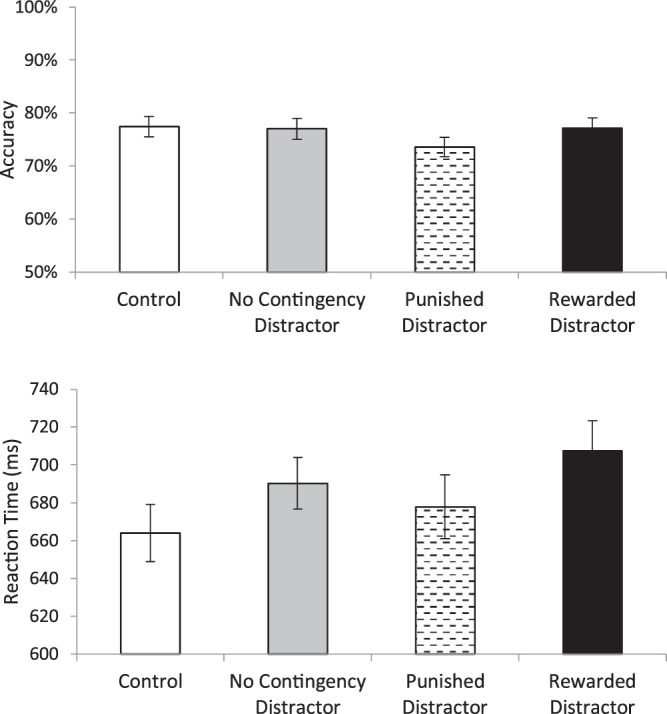
The top panel presents accuracy during the test phase of Experiment 1 as a function of distractor condition. The bottom panel presents reaction time as a function of distractor condition. Error bars represent the standard error of the mean.

Descriptive statistics are presented in Table 1. A repeated-measures ANOVA on accuracy (see Figure 3) with four levels of distractor type (control, no-contingency distractor, punished distractor, and rewarded distractor) found a significant main effect (F(3, 135) = 6.21, *p* = 0.001, η_p_^2^ = 0.12). Paired *t*-testing demonstrated that the rewarded distractor condition had lower accuracy than all other conditions (all t(45) > 3.34, all *p* < 0.003, all d > 0.288 < 0.332). No other conditions differed (all t(45) <.38, *p* > 0.7; BF_01_ > 8.08).

**Table 1:** . Test phase means and within-subject 95% confidence intervals (Loftus & Masson, 1994). *Note:* RT = reaction time.

	Accuracy (95% Conf.)	RT in ms (95% Conf.)
Experiment 1		
Control	0.773 (0.736–0.810)	664 (653–675)
No contingency distractor	0.769 (0.730–0.807)	690 (679–702)
Punished distractor	0.770 (0.731–0.808)	678 (667–689)
Rewarded distractor	0.733 (0.698–0.768)	707 (696–719)
Experiment 2		
Control	0.798 (0.781–0.815)	681 (671–691)
No contingency distractor	0.797 (0.780–0.814)	679 (669–689)
Punished distractor	0.791 (0.774–0.808)	680 (670–690)
Rewarded distractor	0.794 (0.777–0.811)	696 (686–706)

A repeated-measures ANOVA on reaction time with four levels of distractor type (control, no-contingency distractor, punished distractor, and rewarded distractor) found a significant main effect (F(3, 135) = 11.03, *p* < 0.001, η_p_^2^ = 0.20). Paired *t*-test demonstrated that the rewarded distractor condition resulted in significantly longer reaction times than the punished and control condition (all t(45) > 3.91, all *p* < 0.001, d = 0.264 and 0.409, respectively) and was marginally slower than the No Contingency Distractor condition (t(45) = 1.97, *p* = 0.055, d = 0.169). The punished distractor did not differ significantly from the no contingency distractor condition (t(45) = 1.34, *p* = 0.19; BF_01_ = 3.67) and was slower than the control condition (t(45) = 2.17, *p* = 0.035, d = 0.127). The no contingency distractor condition also yielded significantly slower reaction times than the control (both t(45) = 3.43, *p* = 0.001, d = 0.269).

## Discussion

The training phase clearly indicated that participants were sensitive to the reward contingencies. As training progressed, participants learned to rapidly attend to the rewarded targets and to a lesser extent they also learned to rapidly identify the no reward contingency targets. Performance for these two types of targets gradually increased over the five training blocks. However, for the punished target, there was little change in correct identification beyond the second block. In short, our training phase was effective in encouraging people to rapidly attend to rewarded targets but had little effect on punished targets.

More interestingly, in the test phase we found robust value-based capture effects for the rewarded color; the rewarded distractors were more distracting than any other condition. We also found that the no-contingency and punished distractor conditions were more distracting than the control condition in which none of the distractors were target colors during training. This suggests that simply being a target during training imbues that feature with some attentional prioritization and is consistent with prior reports of these target-based effects (Sha & Jiang, 2015). However, in that article, the authors argued that effects attributed to reward contingencies might be entirely due to these target-based effects. The fact that our rewarded condition produced more distraction than the no-contingency distractor condition suggests that reward increases attentional prioritization above and beyond the effect produced by being a task-relevant target during training and is consistent with studies showing that pairing reward with task-irrelevant stimuli can also lead to value-based attention capture (Hopf, Schoenfeld, Buschschulte, Rautzenberg, Krebs, & Boehler, 2015; Le Pelley, et al., 2015; Mine & Saiki, 2015).

Finally, we find that the punished distractor condition is significantly less distracting than the rewarded distractor, and although there is a trend toward being more distracting than the control condition, the punished distractor is clearly no more distracting than the no-contingency distractor. Thus our results suggest that associating attention to a particular color with a loss results in that color neither attracting nor suppressing attention.

However, we note a few potential issues that could weaken the conclusion from Experiment 1. First, it is possible that in the punished condition participants were learning to rapidly attend to the punished color and simply withhold responses. We doubt that is the case for the following reasons. If the participants learned to rapidly attend to the punished distractor and withhold the response, we would expect attention to be captured by distractors that matched the punished color, but during testing we found no evidence of increased slowing by this distractor. In addition, if this was a strategic decision to attend to the punished color and withhold response, then it should have occurred only for those participants who were aware of the contingencies. To investigate this issue, we examined the responses to the open-ended survey question that asked participants to describe why they gained or lost money on a trial. We coded participants who mentioned anything about the color of the stimulus influencing the reward contingencies as explicitly aware. Based on this criterion, 24 of our subjects were explicitly aware of some of the payout contingencies. To assess the contribution of awareness, we ran a mixed-factorial ANOVA on the reaction time during the test phase, with awareness as a between-subject factor and distractor type as a within-subject factor. We found no main effect of awareness (F(1,44) = 1.79, *p* = 0.188, η_p_^2^ = 0.039) nor an awareness by condition interaction (F(3, 132) < 1, *p* = 0.67, η_p_^2^ = 0.012). In short, there was no evidence that the pattern of results in the test phase relied on explicit knowledge of the reward contingencies, thereby casting doubt on the strategic explanation of these results based on rapidly finding the punished color but withholding responses (see Supplementary Material including Supplementary Figures S1, S2, and S3 for more complete analyses of the effect of awareness).

A second potential shortcoming is that training task accuracy was lowest for the loss-associated color condition, which differs from previous studies reporting capture of attention by punishment (Wang, Yu, & Zhou, 2013; Wentura, Müller, & Rothermund, 2014), which reported equivalent accuracies across training conditions. As a result, participants in our experiment experienced fewer trials of negative feedback from the punished color than positive feedback from the rewarded color. This translates to less opportunity for learning in the punished condition. This difference in accuracy during training also resulted in differences in “selection history.” Selection history can influence attention such that items that have been selected in the past are more likely to be selected in the future (Awh, Belopolsky, & Theeuwes, 2012). Thus fewer correct trials during the training phase for the punished color condition raises the possibility that our results during the test phase were due to difference in selection history or the number of learning opportunities rather than reward contingency effects, per se. Similarly, given the way reward contingencies were implemented in Experiment 1, some participants could have adopted an approach to only look for the rewarded color during training, thereby reducing the effects of training for the punished color. To address these shortcomings we performed Experiment 2.

## Experiment 2

The main goal of Experiment 2 was to determine whether the results demonstrating capture by reward but not by punishment would replicate given a design that equated selection history and learning opportunities across conditions. The experiment used a training task similar to Experiment 1's but with an algorithm that dynamically changed the number of trials presented for each condition to ensure an equal number of correct responses across training conditions. If the results of Experiment 1 were simply due to selection history or the number of learning opportunities, we would expect the punished color to attract attention to a similar degree as the rewarded color. If, however, the effects were due to the consequence (reward or loss) associated with a color, the results should replicate those of Experiment 1.

## Method

### Participants

A new group of 46 undergraduates participated for financial compensation; all reported normal or corrected-to-normal vision and normal color vision. Participants gave informed consent and the experimental procedure was approved by the Michigan State University Institutional Review Board.

#### Stimuli

Visual stimuli and computer displays were identical to those in Experiment 1.

### Design and procedure

The method was identical to Experiment 1, except a change in the training phase. Similar to Experiment 1, each trial contained a single target which was associated with either reward, punishment, or no consequence (neutral). Different from Experiment 1, we implemented an adaptive method to equate the number of correct responses across three conditions. The computer program maintained three counters that stored the cumulative number of correct responses for each condition (rewarded, punished, neutral). After each trial, the program determined the condition for the next trial based on the following rules: if the three counters had equal values, then the next trial will be randomly selected from the three trial types with equal probability; if one value was higher than the other two values, then the next trial will be randomly chosen from the latter two types; if two values were both higher than the third value, then the next trial will be the same type as the third type. After participant responded, the counter corresponding to the trial type was updated such that its value was incremented by one if the participant was correct in the line orientation judgment on the current trial. This algorithm guarantees that the total number of correct trials will be at most different by one across conditions during the entire learning phase (see Results below), thus equating selection history across the three conditions.

## Results

We eliminated the data from four participants because their overall accuracy in the reward condition was below 50% for the last four blocks of training, leaving 42 participants for analysis. After this trimming there were 13 participants in the green rewarded, 14 in the red rewarded, and 15 in the yellow rewarded conditions.

### Training phase

**Figure 4.**
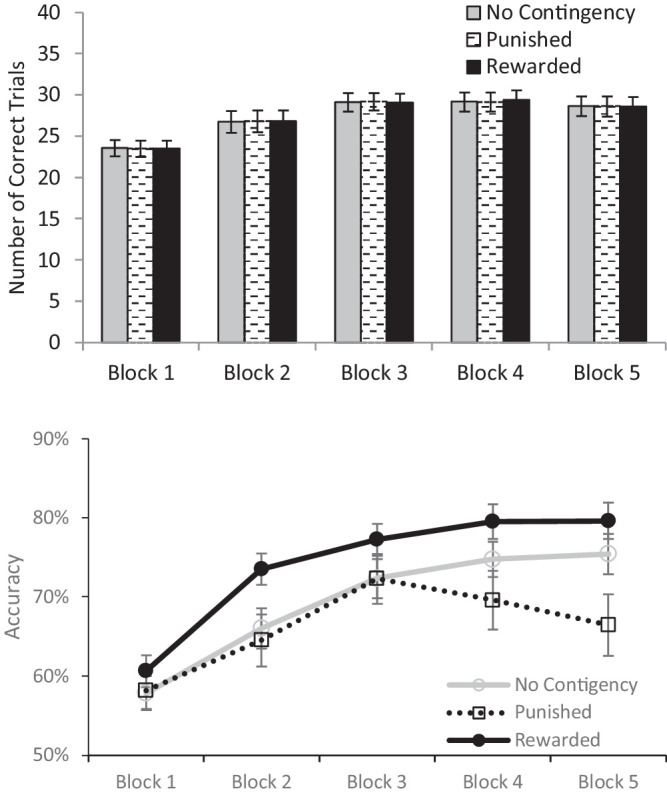
The top panel shows the number of correctly responded trials for the three conditions in each block of the training phase in Experiment 2. The near identical values across conditions show that our algorithm of equating selection history is effective. The bottom panel shows the percentage of times that participants correctly reported each type of target as a function of display type and training block.

Our algorithm was successful at equating the selections of each color; 16 participants had exactly equal numbers of correct responses across the three training conditions, with the remaining 26 varying by only one more correct trial in the highest condition than the lowest. Unsurprisingly, an ANOVA on the number of correct responses for each condition showed that conditions did not differ (F(2, 82) = 0.04, *p* = 0.96, η_p_^2^ = 0.001), and, as Figure 4 (top panel) shows, the average number of correct responses was equated across each condition within each block. However, as with the previous experiments, the percentage correct (see Figure 4, bottom panel) did differ across conditions (F(2, 82) = 7.56, *p* = 0.001, η_p_^2^ = 0.156), with significantly better performance for the rewarded group (M = 74.1%, SE = 1.8%) than either the punished (M = 66.2%, SE = 2.8%) or the neutral (M = 69.3%, SE = 2.0%) conditions, both (t(41) > 3.22, ps < 0.002, ds > 0.39). The punished condition did not differ significantly from the neutral condition (t(41) = 1.37, *p* = 0.178, BF_01_ = 3.37). The reason that the number of correct detections was equated, even though the percentage correct differed, was that the algorithm resulted in significantly (F(2, 82) = 7.71, *p* = 0.001, η_p_^2^ = 0.16) fewer rewarded target trials (M = 187.7, SE = 5.11) than no consequence (M = 201.12, SE = 5.09) or punished (M = 225.5, SE = 10.54) trials (both t (41) > 3.15, p < 0.004, d > 0.40). The number of trials for the punished condition was significantly more than the number of trials for the neutral condition (t(41) = 2.13, *p* = 0.04, d = 0.45). As a consequence of equating the number of correct responses, the net gain/loss during training was close to 0. The final payoff for all participants were essentially constant, in the range of $10 ± 0.1 across conditions.

### Test phase

**Figure 5.**
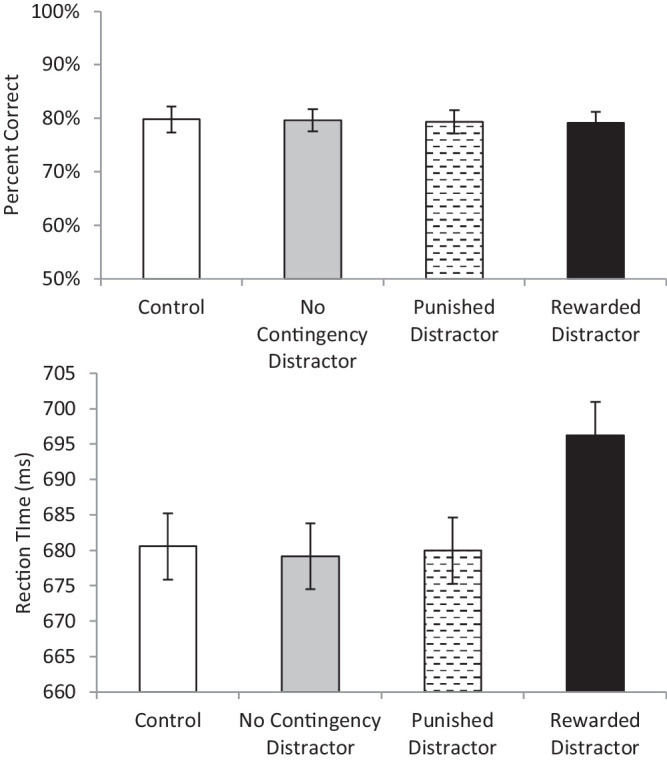
The top panel presents accuracy during the test phase of Experiment 2 as a function of distractor condition. The bottom panel presents reaction time as a function of distractor condition. Error bars represent the standard error of the mean.

Descriptive statistics are presented in Table 1 and Figure 5 presents the mean accuracy and reactions times as a function of condition. An ANOVA on accuracy (F(3, 123) < 1, *p* = 0.93, η_p_^2^ = 0.004; all BF_01_ > 6.88) showed no difference in accuracy across conditions. The reaction time pattern basically replicates that of Experiment 1; there is evidence of the standard reward capture effect but the punished distractor has little effect. An ANOVA on the reaction time data was significant (F(3, 123) = 2.79, *p* = 0.043, η_p_^2^ = 0.064) and the pairwise comparisons showed that the rewarded distractor significantly slowed reaction times relative to the no-contingency distractor condition (t(41) = 2.46, *p* = 0.018, d = 0.152) and the punished distractor condition (t(41) = 2.12, *p* = 0.04, d = 0.138). Furthermore, the no-contingency distractor condition did not differ from the punished distractor condition (t(41) = 0.12, *p* = 0.91; BF_01_ = 8.25). Thus the data pattern replicates those of Experiment 1 with one exception: the control condition that involved a distractor that had not been a target during the training did not differ from the punished and no-contingency distractors (both t(41) < 0.22, *p* > 0.8; BF_01_ > 8.11).

## Discussion

Through a dynamic updating algorithm, we equated the number of correct responses among the three conditions during training. If Experiment 1 failed to show capture by a punished color because the infrequent selection history or limited opportunities for learning were counteracting a bias to attend to punished features, then Experiment 2 should have shown a robust attentional bias for the punished color. Instead, we again found that associating a color with reward resulted in an attentional bias, but associating a feature with loss produced no such bias. Thus we replicated the effects of Experiment 1, and the results of this experiment could not be explained by differences in learning opportunities or selection histories between conditions. We conclude that the failure to find attentional capture by the punished color in these experiments was not caused by fewer selections, but resulted because loss appears not to capture attention. Notably, gains do, even when the selection history is matched with other conditions.

Again, to examine whether the results were driven by intentional, strategic responses among those who became aware (n = 20) of the contingencies, we performed a mixed-factorial ANOVA on the reaction time during the test phase, with awareness as a between-subject factor and distractor type as a within-subject factor. We found no main effect of awareness (F(1, 40) = 2.94, *p* = 0.094, η_p_^2^ = 0.068) and no distractor type by awareness interaction (F(3, 120) < 1, *p* = 0.98, η_p_^2^ = .001). The lack of an awareness by distractor type interaction suggests that the patterns of results we found in the test phase were not due to strategic shifts among those who became explicitly aware of the reward contingencies, but it also occurred for those who lacked this explicit knowledge (see Supplementary Material including Supplementary Figures S1, S2, and S3 for more complete analyses of the effect of awareness).

## General discussion

Across both experiments we found robust evidence that associating attention to a particular color with reward led to subsequent attentional capture by that color, the standard value-driven capture effect. In Experiment 1, this was true even when compared with capture by a color that was a potential target, but had no reward contingency, during training. Although some have argued that the value-driven capture effect may be due to the color being associated with a potential target rather than the reward contingencies (Sha & Jiang, 2015), our results suggest that being a potential target during training can increase attentional capture by that color (at least in Experiment 1), but associating a color with reward increases attentional capture beyond this target-based capture.^1^ Furthermore, our conclusion is consistent with findings that a color associated with high reward produces more attentional capture than a color associated with low reward; in those designs both colors are potential targets, yet the higher rewarded color produces more capture (Anderson, Laurent & Yantis, 2011b).

Besides replicating the standard value-driven capture effect, the main focus of our experiments was to investigate the effect of associating attention to a color with loss rather than reward. Across both experiments we found no evidence that a color associated with loss during training captured attention during the test phase; the target associated with loss was never more attention grabbing than the target that had no reward contingency during training, and was consistently less attention grabbing than the color associated with reward, even when we equated selection history between colors. These observations were supported by the Bayesian analyses which provided consistent positive support for the null hypothesis that the loss-associated condition did not differ from the control conditions. This finding is in direct contradiction to findings that loss-associated stimuli capture attention to the same extent as reward-associated stimuli (Müller et al., 2015; Wang et al., 2013; Wentura et al., 2014). Those authors argued that the “value” in value driven capture is based on the informational relevance of the stimuli since it signals a consequence, rather than the valence of that consequence. As we discussed in the Introduction, in those designs, attending to the punished color reduced loss relative to failing to attend to that color. As a result, paying attention to the loss-associated color was actually rewarded in a relative sense. By contrast, in our experiments, attending to the loss-associated color was punished, while failing to attend to it was not. Thus, our interpretation is that attention is captured by features that are associated with relative gains, but not captured by features associated with loss.

A few other researchers have used quite different methods from ours and have also concluded that gains may attract attention but losses do not. For instance, Raymond and O'Brien (2009) associated faces were with loss or gain during a game in which participants selected one of two faces. After selecting a face, a reward or loss of money (five pence) was delivered. After training, participants performed an attentional blink procedure in which they looked for two targets. Faces that were associated with reward did not suffer from the attentional blink, suggesting that they were prioritized for attention. However, those associated with loss suffered from an attentional blink, suggesting that pairing the face with loss did not increase its attentional priority. An experiment by Gupta, Hur and Lavie (2016) used a similar training game to associate specific neutral faces with gains or losses. After training, the faces appeared as irrelevant distractors when the participant searched an array of letters for a target letter. When the search task was a high perceptual load task that encouraged the filtering of irrelevant stimuli, the face associated with reward captured attention, whereas the face associated with loss did not. Both of these studies are consistent with our findings that value-based capture is specific to reward-associated stimuli. Our results thus provide converging evidence for this conclusion using the standard value-based capture paradigm.

Other researchers have claimed that pairing a feature with loss may actually result in suppression of attention by that feature (Bucker & Theeuwes, 2016; Müller et al., 2015). Our experiment was not designed to detect suppression, and our test phase may have been insensitive to suppression effects, even if the punished distractor was effectively suppressed that would amount to reducing the number of distractors in the display by one item, which may have little impact on the overall reaction time to find the shape target. However, there may be preliminary evidence for the suppression of attention by loss in our training data. In both experiments, participants were less likely to respond rapidly and correctly to the loss-associated target than either the no-contingency or the rewarded target during training. This reduction in accuracy may be a result of suppression of attention by loss. In addition, across both experiments, an ANOVA on the accuracy data for the punished condition as a function of block found a significant quadratic trend (Exp 1: F(1, 45) = 10.14, *p* = 0.003, η_p_^2^ = 0.18; Exp 2: F(1, 41) = 23.33, *p* < .001, η_p_^2^ = 0.36), suggesting an initial increase in correct responses followed by a subsequent decrease as one learns to suppress attention to the punished color. Although this is only weak evidence of suppression, it may be fruitful to conduct future experiments with more sensitive designs to demonstrate a genuine suppression effect.

Although speculative, we can envision two reasons why a reward-associated feature would capture attention in a subsequent test phase, whereas a loss-associated feature would neither capture nor suppress attention during testing. The first is that a single learning mechanism may be responsible for altering the attentional priority of both reward-associated and loss-associated features, but this mechanism may be more sensitive to gains than losses. Under this explanation, the magnitude of gains we used may have been potent enough to drive the learning mechanism, resulting in subsequent attentional capture, but the same magnitude of loss may have been insufficient to drive this learning mechanism, leading to neither capture nor suppression by a loss-associated feature. Results showing that Pavlovian fear conditioning of a stimulus feature results in subsequent capture of attention by that feature may be consistent with this explanation (Armony & Dolan, 2002; Koster, Crombez, Van Damme, Verschuere, & De Houwer, 2005; Schmidt, Belopolsky, & Theeuwes, 2015; Wang et al., 2013). For example, if the negative association is created by pairing a color with an electric shock, the magnitude of the negative consequence may be great enough to drive a value-based learning mechanism, resulting in capture of attention by colors that are associated with negative outcomes.

Although the above scenario is plausible, we favor a second possibility, derived mostly from neurobiological evidence. Namely, that the mechanism responsible for the standard value-based capture effect is driven by rewards but not by losses. Consistent with this proposal are recent suggestions that the capture of attention by reward-associated features is due to increased dopamine (Schultz, Dayan, & Montague, 1997; Zald et al., 2004), perhaps specifically in the striatum (Anderson et al., 2016; Anderson et al., 2017). If the mechanism responsible for learning the reward contingencies is due to the release of striatal dopamine, losses, which produce a reduction of striatal dopamine (Oleson et al., 2012), may not activate this learning mechanism. As such, gains should produce learned associations whereas losses should not. Under this explanation, the results showing attentional capture by Pavlovian fear conditioning would be driven by a completely different mechanism. Fear conditioning is usually associated with neural activity in the amygdala (Fendt & Fanselow, 1999; Maren, 2001; Phillips & LeDoux, 1992) and thus may be subserved by a different system than the striatal dopamine system involved in the standard value-based capture effect. To summarize, we found that associating a color with reward caused attentional capture in a subsequent test phase even when color was no longer relevant. Associating a color with loss, however, resulted in neither capture nor suppression of attention to that color. The distinct effect of reward and loss on attentional prioritization might suggest distinct neural systems responsible for learning via reward that is not activated in response to loss. Further research is necessary to delineate how motivational and emotional outcomes alter attentional priority of stimulus features.

## Supplementary Material

Supplement 1

Supplement 2

Supplement 3

Supplement 4

## Footnotes

1 It remains unclear why Sha & Jiang (2015), failed to find evidence for this type of reward based attentional capture, but it is worth noting their method could have included response conflict (i.e., many distractors were associated with the opposite response as the target) and their overall reaction times were much longer than those in tasks that found evidence for value based capture.
