# Supplementary figures and images for: Value-based attention capture: Differential effects of loss and gain contingencies
Source: J Vis. 2020 May 12;20(5):4. doi: 10.1167/jov.20.5.4 (PMC7409594; doi:10.1167/jov.20.5.4)

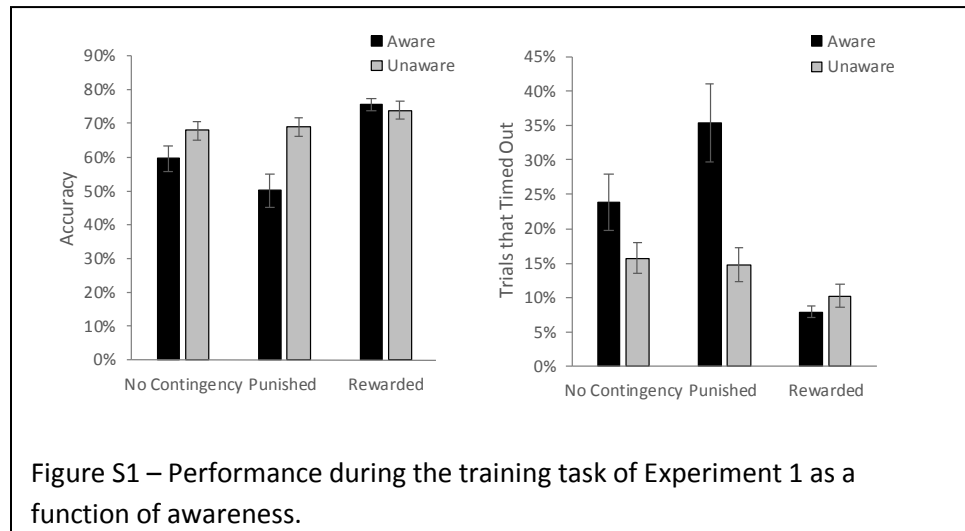

Supplement: Supplement 1 [file jovi-20-5-4_s001.pdf]

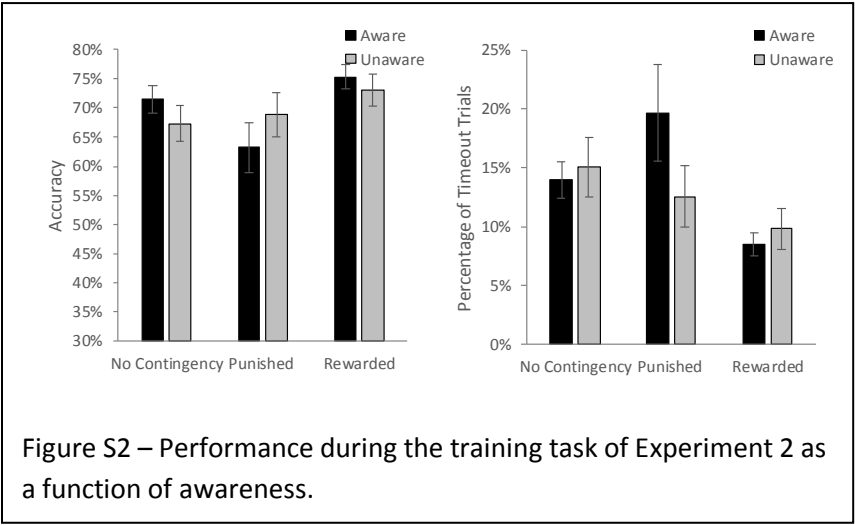

Supplement: Supplement 2 [file jovi-20-5-4_s002.pdf]
