# Supplementary material for: Value-based attention capture: Differential effects of loss and gain contingencies
Source: J Vis. 2020 May 12;20(5):4. doi: 10.1167/jov.20.5.4 (PMC7409594; doi:10.1167/jov.20.5.4)
Supplement: Supplement 3 [file jovi-20-5-4_s003.pdf]

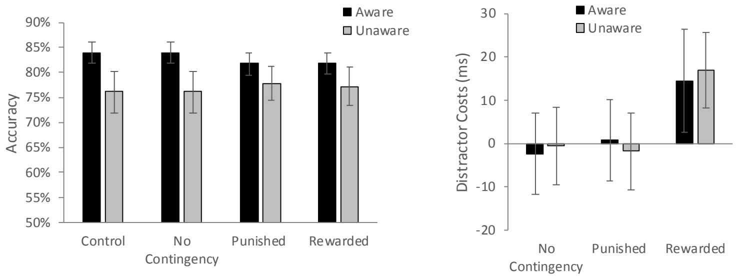

Figure S3 – Performance during the test phase of Experiment 2 as a function of awareness. The distractor costs depicted in the rightward panel were calculated by subtracting the RT for the control condition from the RT for each condition. Error bars are standard errors of the mean.
