# Supplementary material for: Value-based attention capture: Differential effects of loss and gain contingencies
Source: J Vis. 2020 May 12;20(5):4. doi: 10.1167/jov.20.5.4 (PMC7409594; doi:10.1167/jov.20.5.4)
Supplement: Supplement 4 [file jovi-20-5-4_s004.docx]

**Supplementary Materials**

Here we address whether subjective awareness had any impact on our results. We should note that awareness was not the main point of our experiments, and the reliability and validity of self-reported awareness measurements have been questioned (Vadillo, Konstantinidis, & Shanks, 2016; Vadillo, Linssen, Orgaz, Parsons, & Shanks, 2019). Nevertheless, for the sake of completeness, we reanalyzed our data from both the training and test phase as a function of whether participants became aware of the reward contingencies. We present the detailed results of the effect of awareness for each experiment below.

**Experiment 1.**

In Experiment 1, there were 24 participants who became aware of the reward contingency and 22 participants who remained unaware. We first examined performance in the training phase for the aware and unaware participants separately. We ran mixed factorial ANOVAs on accuracy and the number of timeout trials with awareness as a between-subject factor and reward condition as a within-subject factor. For both accuracy and timeout trials there was a main effect of condition [accuracy: F(2, 88) = 19.85, p<.001, η_p_^2^ = .31; timeouts: F(2, 88) = 17.78, p<.001, η_p_^2^ = .29], a main effect of awareness [accuracy: F(1, 44) = 5.43, p = .024, η_p_^2^ = .11; timeouts: F(1,44) = 6.27, p =.016, η_p_^2^ = .125] and an awareness by condition interaction [accuracy: F(2, 88) = 8.39, p <.001, η_p_^2^ = .16; timeouts: F(2, 88) = 8.73, p<.001, η_p_^2^ =.166]. As shown in Figure S1, aware participants were more likely to make errors and have more trials that timed out for the punished color, whereas performance for the no contingency and rewarded colors were more similar between aware and unaware groups. This result likely indicates a level of strategic responding among those aware participants.

___________________

Figure S1 about here

___________________

While differences based on awareness occurred during training, they did not persist into the test block once the reward contingencies had been removed. Mixed factorial ANOVAs on the errors and the reaction times during the test phase found neither a main effect of awareness [accuracy: F(1, 44) < 1, p= .86, η_p_^2^ = .001, reaction time: F(1,44) = 1.79, p =.188, η_p_^2^ = .039] nor an awareness by condition interaction [errors: F(3, 132) <1, p=.89, η_p_^2^ = .005; reaction time: F(3, 132)< 1, p=.67, η_p_^2^ = .012]. In sum, while awareness may lead to differences in response strategies during the training phase, both aware and unaware groups showed the same pattern of responses during the test phase, as shown by Figure S2. Thus, it appears that the persistence of the attentional influence of the reward contingencies is isolated to an increased attention bias toward rewarded colors, and this persistence does not depend on explicit awareness.

___________________

Figure S2 about here

___________________

**Experiment 2.**

In Experiment 2, there were 20 participants who became aware of the reward contingency and 22 participants who remained unaware. We first ran mixed factorial ANOVAs on accuracy and the number of timeout trials with awareness as a between factor and reward condition as a within factor for the training phase. There were main effects of condition [accuracy: F(2, 80) =8.32, p = .001, η_p_^2^ =.172; time outs: F(2, 80) =8.65, p <.001, η_p_^2^ = .178], no main effects of awareness [accuracy: F(1, 40) <1, p = .96, η_p_^2^ <.001; time outs: F(1, 40) <1, p = .058, η_p_^2^ = .008], but there were significant awareness by condition interactions [accuracy: F(2, 80) = 3.38, p = .039, η_p_^2^ = .078; time outs: F(2, 80) = 3.74, p = .028, η_p_^2^ = .086]. Similar to Experiment 1, it appears that source of the interaction was that only aware participants showed lower accuracy and higher timeouts for the punished color (see Figure S3). Again, this suggests that aware participants were strategically avoiding the punished color or withholding responses.

___________________

Figure S3 about here

___________________

Again, similar to Experiment 1, the effect of awareness was confined to the training phase. Mixed factorial ANOVAs on the accuracy and the reaction times during the test phase found neither a main effect of awareness [accuracy: F(1, 40) =11.53, p= .22, η_p_^2^ = .037, reaction time: F(1,40) = 2.94, p =.094, η_p_^2^ = .068] nor an awareness by condition interaction [accuracy: F(3, 120) =1.30, p =.28, η_p_^2^ = .031; reaction time: F(3, 120)< 1, p=.98, η_p_^2^ = .001]. As shown in Figure S4, both aware and unaware participants had similar distractor cost during the test phase. In terms of accuracy, it appears that the aware group generally performed better than the unaware group during the test phase, however this did not approach statistical significance.

The effects of awareness is thus consistent across both experiments: the pattern of results suggests that awareness may lead to differences in response strategies during the training phase. However, those strategic differences do not persist once the reward contingencies are removed in the test phase, such that both aware and unaware groups showed the same pattern of responses during the test phase. Thus, regardless of awareness, it appears that rewarded features exert a capture bias that persist into the test phase. However, the avoidance of the punished color does not imbue the associated color with attentional capturing effects in the test phase.

**References**

Vadillo, M. A., Konstantinidis, E., & Shanks, D. R. (2016). Underpowered samples, false

negatives, and unconscious learning. *Psychonomic Bulletin & Review*, *23*(1), 87-102.

Vadillo, M. A., Linssen, D., Orgaz, C., Parsons, S., & Shanks, D. R. (2019). Unconscious or

underpowered? Probabilistic cuing of visual attention. *Journal of Experimental Psychology: General*.
